# Predictive Value of Upper Extremity Outcome Measures After Stroke—A Systematic Review and Metaregression Analysis

**DOI:** 10.3389/fneur.2021.675255

**Published:** 2021-06-10

**Authors:** Silke Wolf, Christian Gerloff, Winifried Backhaus

**Affiliations:** Experimental Electrophysiology and Neuroimaging (xENi), Department of Neurology, University Medical Center Hamburg-Eppendorf, Hamburg, Germany

**Keywords:** stroke, motor rehabilitation, motor recovery, upper limb, motor assessments, motor function, metaregression

## Abstract

A better understanding of motor recovery after stroke requires large-scale, longitudinal trials applying suitable assessments. Currently, there is an abundance of upper limb assessments used to quantify recovery. How well various assessments can describe upper limb function change over 1 year remains uncertain. A uniform and feasible standard would be beneficial to increase future studies' comparability on stroke recovery. This review describes which assessments are common in large-scale, longitudinal stroke trials and how these quantify the change in upper limb function from stroke onset up to 1 year. A systematic search for well-powered stroke studies identified upper limb assessments classifying motor recovery during the initial year after a stroke. A metaregression investigated the association between assessments and motor recovery within 1 year after stroke. Scores from nine common assessments and 4,433 patients were combined and transformed into a standardized recovery score. A mixed-effects model on recovery scores over time confirmed significant differences between assessments (*P* < 0.001), with improvement following the weeks after stroke present when measuring recovery using the Action Research Arm Test (β = 0.013), Box and Block test (β = 0.011), Fugl–Meyer Assessment (β = 0.007), or grip force test (β = 0.023). A last-observation-carried-forward analysis also highlighted the peg test (β = 0.017) and Rivermead Assessment (β = 0.011) as additional, valuable long-term outcome measures. Recovery patterns and, thus, trial outcomes are dependent on the assessment implemented. Future research should include multiple common assessments and continue data collection for a full year after stroke to facilitate the consensus process on assessments measuring upper limb recovery.

## Introduction

From the time stroke patients enter the emergency care unit to the time they return home again, they have stayed in a series of different wards and clinics specialized in various recovery stages. This clinical recovery process is usually well-documented and quantified using a diverse spectrum of scales and assessments as subjective and objective outcome measures. This diversity enables clinicians to describe multiple different patient-specific aspects of the remaining symptoms, but it can also render comparisons across different stroke trials difficult or even impossible. Focusing on subsets of scales tends to lead to an incomplete description of individual recovery profiles. A consensus paper on general recommendations for stroke rehabilitation measurements concluded that many measures are inappropriate to measure recovery (1). The term “recovery,” commonly used in synonym with “motor recovery,” should describe true neurological repair and restitution (2). Both terms reflect the achievement to regain a near-similar state as prestroke concerning body structure and functions and activities of daily living (2–4). Levin et al. (4) stress the importance of the way recovery is achieved, by differentiating between motor recovery and motor compensation. The first is the restoration of function and performance in the same manner as before the incidence of stroke (motor recovery) opposing the recruitment of new tissue or effectors to reach the same goal (motor compensation) (2, 4). Such differentiation can potentially be attained by combining kinematic measures and clinical assessments (2). However, kinematic methods are resource-intensive, costly, and often not available in clinical reality. Although more frequently used, they still fall far short of the established scores [such as the Fugl–Meyer Assessment (F-M)] (5).

Rehabilitation is closely related to recovery and compensation. It is defined as “a process of active change by which a person who has become disabled acquires the knowledge and skills needed for optimum physical, psychological, and social function” (6). Rehabilitation may include improvement in body function going beyond the initial (prestroke) capabilities, a phenomenon that is present especially during later time points after stroke. The recommended time points of data acquisition have been inferred from biological processes following a stroke and comprise five time windows of recovery (2, 3). These include the hyperacute stage (0–24 h), the acute stage (1–7 days poststroke), the early subacute stage (7 days to 3 months after stroke), the late subacute stage (3–6 months after stroke), and the chronic stage for all time points beyond 6 months (2, 3). The initial months after a stroke are subject to multiple biological processes and thus described by three different time windows. A jitter in time of initial study recruitment (length of time since stroke onset) of individual studies can substantially influence its prognostic accuracy (2, 7). This is especially problematic in small and less powered studies (8). The recovery window also seems to exceed the previously defined limits, which further adds to the need for functional measurements (9). A meaningful core set of measurements targeting recovery of hand and arm function should represent all stages, from acute to chronic.

Here we make an attempt to disentangle scoring from common assessments after stroke and illustrate how they measure rehabilitation of upper limb motor function, in the following referred to as “recovery,” over time. With a main objective to assist the decision making process which outcome measures to choose in stroke rehabilitation and to increase the comparability of stroke studies, the aim is less to present the current literature but mainly to extract data from large comparable studies in the field, here defined as studies including at least 100 stroke patients. Methods of a systematic literature review are applied to check the quality of the included papers and to report the process in a transparent and comprehensible way. The following metaregression aims to illustrate the predictive utility of outcome measures most commonly used in stroke rehabilitation studies, focusing on motor recovery, especially upper limb control. Most stroke patients suffer from motor disorders, mainly affecting the arm and hand function, and for many, the impairment is persisting (10, 11). Upper limb rehabilitation is crucial for almost all activities of daily living. Additionally, it stands in close relationship to walking abilities (12). We hypothesized that, by summarizing results from well-powered studies, we could identify those assessments capable of showing change over time, detect redundancies between measures, and provide a basis for recommendations in longitudinal stroke rehabilitation studies.

## Methods

### Search Strategy and Study Selection

**Figure 1:**
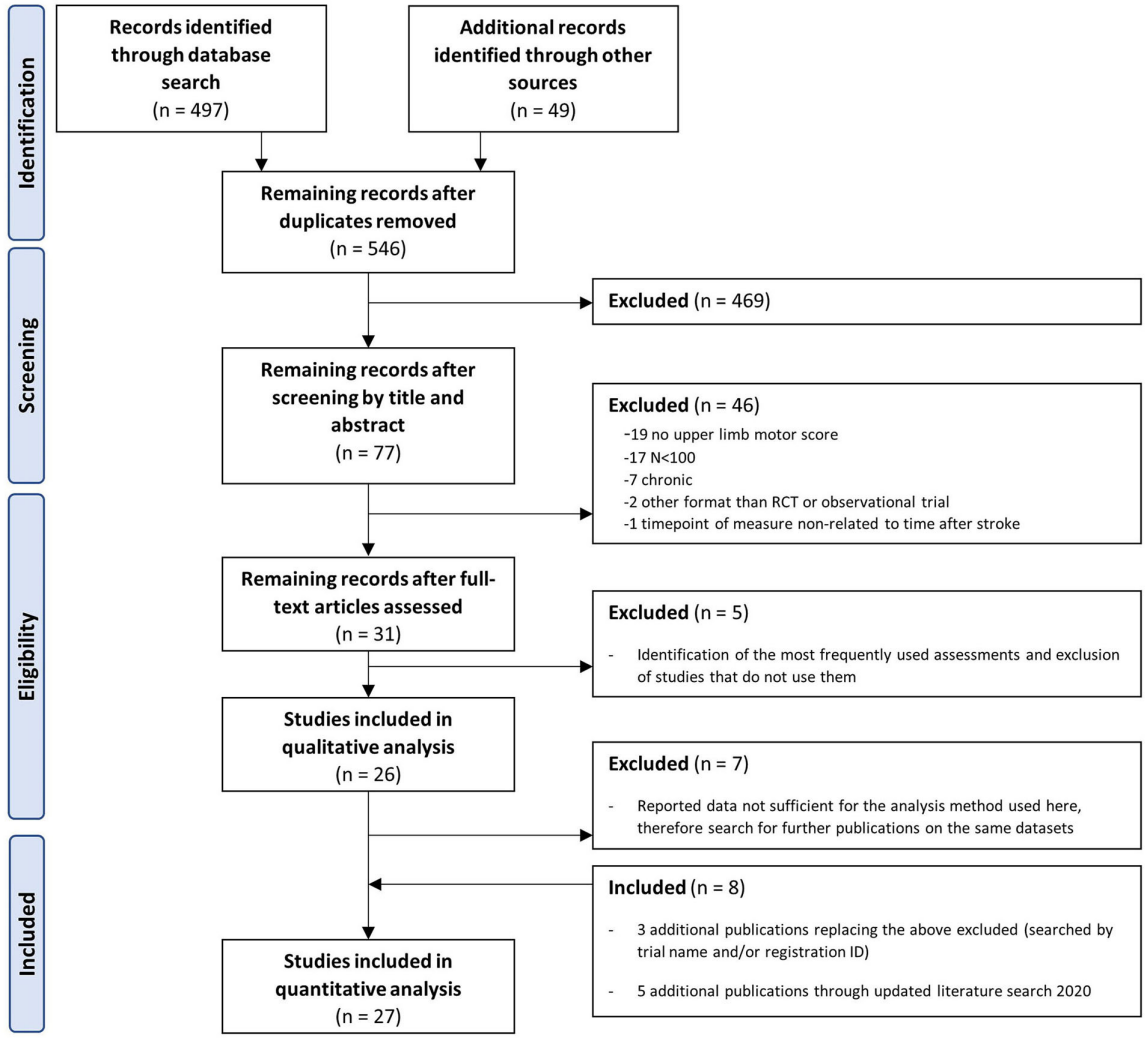
Prisma flow diagram displaying the literature search and eligibility checking process.

Following up on Pollock and colleagues' Cochrane Review (13), a systematic literature search was performed *via* PubMed, updating these findings and addressing how upper limb recovery is quantified in individuals after stroke. The initial search results (May 2018) were updated in November 2020. The search strategy was developed according to the PICOS scheme and included interventional, as well as observational trials performed in adult stroke patients with at least one outcome measure assessing motor function of the upper limb (PICOS: Patients: stroke, adults, 1st year after stroke; Intervention/Comparison: any; Outcome: upper limb motor assessment, hand function; Study/Setting: large (*n* ≥ 100), randomized clinical trials or observational studies, full search query; see Supplementary Materials). Gray literature and further publications based on identical data sets were searched individually, as many publications of large trials do not report the complete data set. The search was done by study name or registration number in trial registration platforms. We restricted the search to publications in English or German language, without restrictions on who performed the outcome measures, whether it was a trained clinician or not. The initial scanning of abstracts of all identified studies and assessments of full papers was done independently by two reviewers (W.B. and S.W.) using the Covidence systematic review software (Veritas Health Innovation, Melbourne, Australia). The remaining articles included only well-powered studies, with at least 100 participants reporting upper extremity motor assessments (for flowchart, see Figure 1). Between-rater differences in interpretation of the study data were resolved by discussion. The subsequent selection of assessments was based on their frequency of use. For data analysis reasons, only those assessments for which data were available for at least two different time points in at least two different studies could be included. Quality assessment of randomized trials was conducted by S.W. applying the revised Cochrane risk-of-bias tool for randomized trials (14). The tool judges the overall risk of bias on the basis of five domains: selection of the reported results, measurement of the outcome, missing outcome data, deviation from intended interventions, and randomization process. Judgment can be “low” or “high” risk of bias or “some concerns” each represented by green, red, or yellow color, respectively (14). The publication quality did not result in data exclusion or the weighting of data points.

### Data Extraction and Preprocessing

Population characteristics and assessments with their respective means and standard deviations (SDs) were extracted for each study arm relative to the time point after stroke within each study population. In the case of incomplete information, the respective authors were contacted by mail at least twice. In some cases, the original raw data were no longer available or at hand. Values provided as medians and range values were transformed to means and SD (15) if the author could not provide the means and SD otherwise. Also, further publications relying on identical data sets were searched for and screened for additional information. We excluded some studies or individual outcome measures within studies due to a lack of information (Supplementary Table 1). The final data are hierarchically structured, including multiple studies, each with multiple intervention groups and multiple assessments measuring motor function at multiple time points, depicted in weeks or months, within the first year after a stroke. For more straightforward comparability and interpretability, scores of all assessments were rescaled from their original scale to a standardized recovery score ranging from 0 to 100, the latter being fully recovered. The rescaling was done relative to the available range of the individual assessment. To receive a full recovery score, individuals required 66 points on the F-M (upper extremity) or should be as fast as 1.3 s on the Wolf Motor Function Test (performance time WMFT; Table 1). In cases where multiple outcomes were measured simultaneously within one study, the standardized score will still differ between the individual outcomes. Outcomes measured in the 10- and 9-hole peg tests were rescaled to “pegs per second” and merged to one “peg test” (PEG) before standardization. Intervention and control groups were reassigned to experimental and standard therapy but not merged to avoid potential loss of information. In the case of multiple intervention groups, this could result in multiple, identically coded, experimental, or standard therapy groups per study and time point. During this data pooling process, there was a deliberate decision not to model the effect of the different interventions. The included studies represent the variety of methods used in neurorehabilitation. A quality assessment of these methods is not the subject of the present study. Quality evaluation of evidence for interventions is the subject of other work (13).

**Table 1:** Rescaling of assessments to their respective percentage of recovery.

**Assessment**	**0% recovery**	**100% recovery**
Action Research Arm Test	0 points	57 points (16)
Box and Block test	0 blocks/min	74 blocks/min (17)
Fugl–Meyer Assessment, upper limb	0 points	66 points (18)
Grip force (ratio affected/non-affected hand)	0	≥1
Motricity Index	0 points	100 points
Peg test (9-hole peg test and 10-hole peg test)	0 pegs/s	2.3 pegs/s (19)
Rivermead Motor Assessment	0 points	15 points (20)
Stroke Impact Scale—hand function	0 points	25 points (21)
Wolf Motor Function Test	120 s	1.3 s (22)

### Statistical Analysis

A metaregression analysis was performed in R (version 3.5.0) (23) within the metafor package (version 2.1-0) (24) with the goal to illustrate how different assessments map recovery over time. As recovery is not a linear process but shows more change during the initial months, we chose to calculate effect sizes using the log-transformed mean (MNLN). For mathematical reasons, data with SDs equal to zero had to be removed. This concerned a total of 10 data points measuring Action Research Arm Test (ARAT) (25), WMFT (26), and PEG (27) at 1 and 3 weeks after stroke. As a reference group, we chose to add hypothetical “healthy” scores with a mean of 98 and SD of 2 on the standardized score. This value for mean and SD was chosen based on the included assessments and their respective score for healthy controls [i.e., full upper limb capacity in the ARAT = 55–57 points (28), which is equivalent to 96.5–100%]. The reference group's size was identical to the compared study population at the selected time point.

The studies from which data were collected pursued multiple different research goals; a random-effects, multivariate metaregression (24) acknowledges these differences. Time (weeks or months after stroke), assessment (categorical: healthy, ARAT, F-M, etc.), and estimates within studies were added as random terms to reflect the hierarchical structure and differences between studies and assessments. Restricted maximum likelihood was used as a model estimator. An unstructured variance–covariance matrix was implemented to allow differences in variances and correlations within the random effects. The initial model included the factor ASSESSMENT used to measure recovery, a logarithmic term of the TIME after stroke to account for the data spread, a linear term of TIME after stroke, the interaction of the latter with ASSESSMENT, and a term for “intervention group,” added to account for possible heterogeneity. The Glmulti package (29) assisted model selection using maximum likelihood estimation. The final model was subsequently utilized to predict recovery over 1 year for each assessment. To account for lacking data, especially after 7 months, an additional analysis was performed with the last observation, per study and assessment, carried forward (LOCF). Values from week 7 to week 48 were carried forward to week 52 only if no data were available for week 48 or higher (imputed values *n* = 45). The α level of significance is set to 0.05.

## Results

### Study Selection and Data Extraction

An initial systematic PubMed search identified 497 applicable studies. Additional 49 studies were identified by a manual search for studies relying on identical data sets; references of a recent Cochrane Review (13) were searched for further studies meeting the inclusion criteria. Eligibility screening of abstracts and full text was performed independently by two reviewers (S.W., W.B.). Twenty-seven articles complied with the inclusion criteria (Figure 1). Of these, the motor assessments were extracted, and the frequency of use was assessed. The most frequently used scales included F-M (*n* = 15 studies), ARAT [*n* = 14 (30)], Stroke Impact Scale (SIS, hand score, *n* = 5), Box and Block test (BBT, *n* = 4), WMFT (*n* = 4), grip force (GRIP, *n* = 4), PEG (nine-hole peg test *n* = 2, 10-hole peg test, *n* = 2), Rivermead Motor Assessment (RMA, *n* = 3), and the Motricity Index (MI, *n* = 3) (Table 2). Only assessments including data from at least two different time points in at least two different studies were included in all the following analyses, leading to a final data set based on scores from 4,433 stroke survivors. One study (31) was excluded because its results were based on a data set already included in a previous analysis (26). Additional characteristics of included studies are described in Table 3.

**Table 2:** Frequency overview of the number of studies applying the assessment during at least one time point.

**Assessments**	**No. of studies**
**Included**	
Fugl–Meyer Assessment	15
Action Research Arm Test	14
Stroke Impact Scale—hand function	5
Box and Block test	4
Peg test (pegs/s)^*^	4
Wolf Motor Function Test	4
Grip force	4
Rivermead Motor Assessment	3
Motricity Index	3
**Excluded**	
Range of motion—active	2
Arm motor ability test	2
Motor activity log	2
Motor activity log	1
Motor Assessment Scale	1
Active hand function	1
Profiles of recovery	1
Functional reach	1
Frenchay Arm Test	1
Canadian Occupational Performance Measure	1
Measure of manual ability	1
Hand function through timed manual dexterity performance	1
Motor Club Assessments	1
Adult Assisting Hand Assessment Stroke	1
Chedoke–McMaster	1

*Included: data had to be available from at least two different studies and described for at least two different time points to be included in the metaregression*.

* *Results from different variants of the peg test (9-hole peg test n = 2, 10-hole peg test n = 2) were transformed to pegs per second and summarized as one “peg test.”*

**Table 3:** Characteristics of included studies (alphabetical order).

**References**	**Participants, n (female/male)**	**Mean age (SD), years**	**Trial name**	**Outcome measures**	**Time points of measure**	**Intervention groups (*n*)**	**Baseline/inclusion definition (mean time after stroke at baseline)**
Adie et al. (32)	235 (104/131)	67.4 (13.2)	TWIST	ARAT, SIS	2, 3, 9 months after stroke	Arm exercises, Wii	Up to 6 months after stroke; baseline mean: 2 months after stroke
Brunner et al. (33)	120 (43/77)	62 (NA)	VIRTUES	ARAT, BBT	1, 2, 6 months after stroke	Standard therapy, virtual reality rehabilitation training	Within 12 weeks after stroke
Chen et al. (34)	250 (102/148)	63.3 (10.6)	—	F-M	0, 1, 2 months after stroke	Standard therapy, acupuncture	2–7 days after stroke
Cramer et al. (35)	133 (60/73)	67.7 (11.6)	—	BBT	0, 3 months after stroke	Placebo, monoclonal antibody GSK249320	Within 72 h after stroke
Feys et al. (36)	100 (41/59)	64.2 (11.9)	—	F-M	1, 2, 3, 6, 12 months after stroke	Standard therapy, sensorimotor stimulation (additional)	2–5 weeks after stroke
Ghaziani et al. (37)	102 (52/50)	71.5 (15.5)	—	BBT, F-M, GRIP	7 days, 1, 6 months after stroke	Electric somatosensory stimulation: high dose, low dose	First 7 days
Gialanella and Santoro (38)	208 (101/107)	69.7 (10.3)	—	F-M	1, 2 months after stroke	Standard therapy	All consecutive patients admitted to the Rehabilitation Unit (17 ± 5.4 days)
Guo et al. (39)	120 (53/67)	68.1 (11)	—	F-M	3,4,6,12 months after stroke	MT, ESWT, MT +ESWT, standard therapy	3 months
Harvey et al. (40)	199 (130/69)	59.2 (13)	NICHE	ARAT, F-M, WMFT	Data made available for each month after stroke	Motor training protocol using a navigated brain therapy device, sham	3–12 months after stroke
Ietswaart et al. (41)	121 (51/70)	67.4 (14.3)	—	ARAT, GRIP	3, 4 months after stroke	Standard therapy, motor imagery training, attention-placebo	Within 6 months after stroke
Kong et al. (42)	105 (28/77)	57.6 (11.4)	—	ARAT, F-M, SIS	0, 1, 2, 4 months after stroke	Standard therapy, Wii gaming, control	2 weeks after stroke
Kwakkel et al. (25)	101 (58/43)	65.9 (11.5)	—	ARAT	0, 2, 3, 5, 6 months after stroke	Control, leg training focus, arm training focus	14 days after stroke
Kwakkel et al. (26)	159 (63/96)	60.4 (12.2)	EXPLICIT	ARAT, F-M, SIS, WMFT	0, 1, 2, 3, 6 months after stroke	Usual care, EMG-NMS, mCimt	Within the first 2 weeks after-stroke
Lincoln et al. (27)	282 (138/144)	71.6 (11.5)	—	ARAT, GRIP, PEG, RMA	1, 2, 3, 6 months after stroke	Standard therapy (RPT), standard therapy + PT assistant (APT), standard therapy + qualified personnel (QPT)	1–5 weeks after stroke
Lohse et al. (43)	220 (93/127)	59.8 (12.6)	—	ARAT	Data made available for each month after stroke	Standard therapy	Retrospective analysis
Meyer et al. (44)	122 (45/77)	66.4 (12.4)	—	ARAT, F-M, MI	Data made available for months 1–6 after stroke	Standard therapy	12 days to 6 months after stroke
Morris et al. (45)	106 (45/61)	67.8 (11.5)	—	ARAT, PEG, RMA	1, 3, 6 months after stroke	Unilateral training 6 weeks, bilateral training 6 weeks	2–4 weeks after stroke
Nadeau et al. (46)	408 (184/224)	63.8 (8.5)	LEAPS	F-M	3, 9 months after stroke	Analysis of LEAPS trial (locomotor experience applied poststroke, Duncan et al., (47)—measures at 12 months	Within 45 days after stroke
Opheim et al. (48)	117 (51/66)	69.3 (13.1)	SALGOT	F-M	1, 12 months after stroke	Observation	3 days after stroke
Rodgers et al. (49)	123 (65/58)	74.5 (-)	—	ARAT, MI	0, 3, 6 months after stroke	control—stroke unit care 6 weeks, intervention—enhanced upper limb rehabilitation 6weeks	Within 10 days after stroke
Rodgers et al. (50)	161 (64/97)	60.6 (13.4)	RATULS	ARAT, F-M	5,5 months after stroke	Robot-assisted training, enhanced upper limb therapy, control (usual care)	Up to 5 years after stroke
Saposnik et al. (51)	141 (47/94)	62 (12.5)	EVREST	BBT, GRIP, SIS, WMFT	1, 2, 3 months after stroke	Recreational therapy, virtual reality Wii	Within 3 months after first stroke
van Vliet et al. (52)	120 (60/60)	74.2 (9.8)	—	PEG, RMA	0, 1, 3, 6 months after stroke	Movement science-based treatment, Bobath based treatment	Within 2 weeks after stroke
Veerbeek et al. (53)	202 (106/96)	66.6 (14)	EPOS	F-M	0, 6 months after stroke	Observation	Within 72 h after stroke
Wang et al. (54)	134 (47/87)	56.7 (7.8)	—	F-M	3, 4, 5, 6 months after stroke	Treatment, control	30–90 days after stroke
Wilson et al. (55)	122 (51/71)	56.4 (13.1)	—	F-M	3, 6, 7, 8 months after stroke	Sensory stimulation, 8 weeks, EMG-triggered NMES, 8 weeks, cyclic NMES, 8 weeks	Within 6 months after stroke
Wolf et al. (56)	222 (80/142)	62.1 (13.1)	EXCITE	WMFT	6, 7 months after stroke	CIMT −14 days, usual care	Within 3–9 months after stroke
Total	4,433 (44.3% female)	65 (12)					

*There were 4,433 stroke survivors assessed with various scales at multiple time points. APT, assistant physiotherapist; ARAT, Action Research Arm Test; BBT, Box and Block test; EMG, electromyography; EMG-NMS, electromyography-triggered neuromuscular stimulation; ESWT, extracorporeal shock wave therapy; F-M, Fugl–Meyer Assessment (upper extremity); GRIP, grip force; mCimt, modified constraint-induced movement therapy; MI, Motricity Index; MT, mirror therapy; NMES, neuromuscular electrical stimulation; PEG, peg test; QPT, qualified physiotherapist; RMA, Rivermead Motor Assessment; RPT, routine physiotherapy; SIS, Stroke Impact Scale; WMFT, Wolf Motor Function Test*.

### Study Population and Data Quality

Only data from those groups of individuals with a mean time after stroke within 1 year of the incident were included, the majority of these patients being after ischemic infarction (Supplementary Table 2). The mean age of all participants' subgroups was 65 ± 12 years, with 55.7% being male. The included studies had measured ≥1 of the mentioned assessments at ≥2 time points. A dropout rate per study and time point is presented in Supplementary Table 3.

The risk-of-bias assessment (14, 30) on the methodological quality of the included randomized trials was performed only in those outcomes from which the final data for the current analysis were extracted, even when results were reported in multiple publications. Even if the methodological quality of the papers found is not primarily to be assessed here, the scale serves to assess the bias probability and provides an overview of the current study quality. Overall, a low to moderate risk of bias was observed for the included outcomes (Supplementary Figure 2). Some concerns were documented for the selection of the reported results and regarding the measurements of the outcome. The results of the evaluation did not influence the data used for the analysis.

### Description of Assessments

The final set of outcome measures included the ARAT, BBT, F-M, grip force, MI, peg test, RMA, SIS, and the WMFT (Table 4). This data set included measures as early as within the first week after stroke up to 1 year after the initial symptom onset. Overall, data were available, especially for the first half year after stroke (Supplementary Tables 4, 5).

**Table 4:** Most common assessments used in large stroke trials.

	**Description (57)**
ARAT	Consists of 19 items, ratable on a four-point ordinal scale, ranging from 0 = no movement possible to 3 = normal performance of the task, maximum possible score: 57 points, observer-rated
BBT	A performance-based measure of gross manual dexterity where the patient is instructed to move as many wooden blocks (2.5 cm) as possible from one compartment to another during the time course of 1 min
F-M	The section motor function of upper limb is one of five domains, a three-point scale is used for rating performance as 0 = cannot perform, 1 = performs partially and 2 = performs fully, maximum possible score: 66 points, observer-rated
GRIP	Grip force in different types of grips is tested by using either a dynamometer or a pinch gauge; grip is repeated three times, and averages were calculated maximum grip force ratio was calculated by dividing force affected hand by force healthy hand
MI	A measure of general motor function. The patient is instructed to move and hold joints of upper and lower limb against resistance; muscle force is graded from 0 = no movement to 33 = normal power (58, 59)
PEG	Peg test: a timed measure of fine manual dexterity where the patient is instructed to first take 9 or 10 pegs out of a container and place them into empty holes and back into the container as quickly as possible
RMA	The measurement was developed to assess stroke patients during their course of recovery and includes 38 items on three categories of functional movement: 13 items measuring gross function, and 15 and 10 items, respectively, representing hand and leg function; the rating is dichotomized in 0 = inability to perform and 1 = patient can perform the activity
SIS	Measurement of subjective stroke-specific health status, 64 items in eight domains; domain scores range between 0 and 100, with higher scores representing better health status, self-completed (or face-to-face)—in this work, only hand function is analyzed in more detail
WMFT	Consists of 17 (or 15—short version) items, assessed for performance time (truncated to 120 s) and quality of movement. Only performance time was analyzed in this work

*ARAT, Action Research Arm Test; BBT, Box and Block test; F-M, Fugl–Meyer Assessment; MI, Motricity Index; RMA, Rivermead Motor Assessment; SIS, Stroke Impact Scale; WMFT, Wolf Motor Function Test*.

### Metaregression

An initial model comparison determined which time frame could capture the change of recovery in the available data best: weeks, months, or “5 phases” (hyperacute to chronic) (2, 3) after stroke. Time after stroke in months or weeks represented the factor “TIME” best. As there was no significant difference between models with either measure, determined via Akaike information criterion (AIC) (AIC_months_ = 661.1, AIC_weeks_ = 667.7), the more fine-grained unit, weeks after stroke, was chosen for all further analyses. The fixed-effect “intervention group” and random term for TIME were excluded from the final model, as they did not add to the explained variance. The final model included the moderators ASSESSMENT (nine assessments and “healthy” as the reference category), a logarithmic term of TIME in weeks after stroke, and the interaction TIME × ASSESSMENT. The omnibus test of moderators (QM) yielded a significant result (QM = 228.2, *df* = 20, *P* < 0.001), indicating that all included moderators account for a relevant amount of heterogeneity. The factor ASSESSMENT itself was also a meaningful regressor within the model (QM = 86.7, *df* = 9, *P* < 0.001). On *post-hoc* testing, the main effects of all levels of ASSESSMENT (ARAT, BBT, F-M, GRIP, HEALTHY, MI, PEG, RMA, SIS, WMFT) were significant (*P* < 0.001). The interaction (TIME × ASSESSMENT) enhanced the model significantly [χ^2^(9) = 45.6, *P* < 0.001].

**Figure 2:**
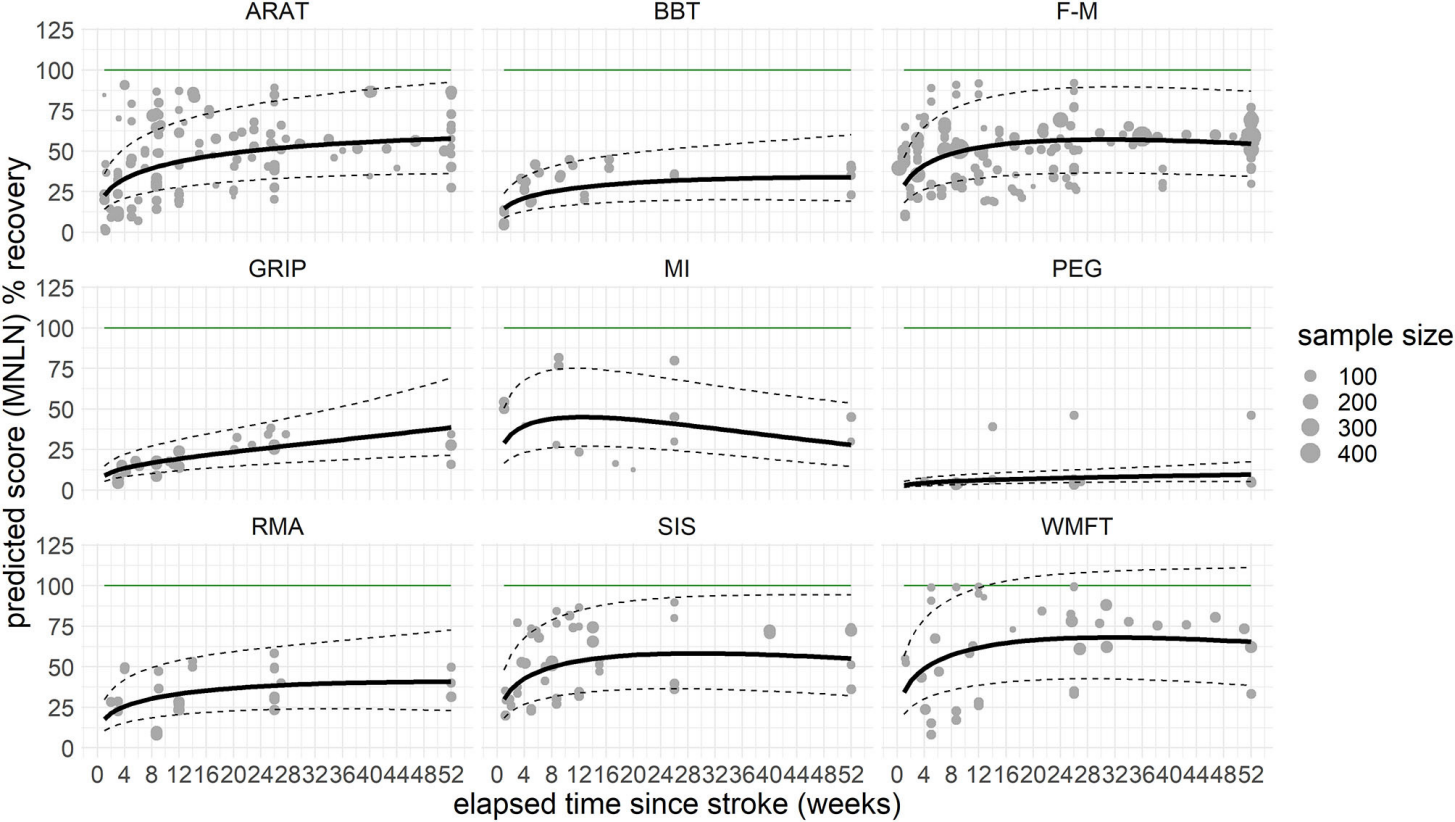
Last observation carried forward (LOCF) prediction of recovery per assessment over the time course of 1 year, cross-fading the underlying raw values. The solid black line represents the predicted recovery pattern, based on log-transformed difference in means (MNLN) within the confidence interval of the prediction (dashed lines). The green horizontal line resembles a healthy score, respective to 100% recovery. The gray dots represent the underlying data points, with the size depicting the sample size of the respective data point. How these underlying data points are related is presented in Supplementary Figure 1. ARAT, Action Research Arm Test; BBT, Box and Block test; F-M, Fugl–Meyer Assessment (upper extremity); GRIP, grip force; MI, Motricity Index; PEG, peg test; RMA, Rivermead Motor Assessment; SIS, Stroke Impact Scale; WMFT, Wolf Motor Function Test.

Not all assessments reached significance levels in the interaction with time, some even showing a downward trend after the initial positive slope of recovery. Only the interactions of TIME with selected assessments, ARAT [χ^2^(1) = 16.5, *P* < 0.001], BBT [χ^2^(1) = 4.2, *P* = 0.040], F-M [χ^2^(1) = 7.5, *P* = 0.006], GRIP [χ^2^(1) = 28.9, *P* < 0.001], and WMFT [χ^2^(1) = 6.4, *P* = 0.011] yielded significant changes, indicating that theses scales are sensitive enough to measure recovery over 12 months. Predicted improvements within both scales BBT and GRIP indicated performance of the stroke-affected hand going beyond the performance of the non-affected hand, a finding possibly linked to handedness (incomplete overview Supplementary Table 2) or lacking data points during the chronic phase after stroke. An LOCF analysis, added to rule out artifacts related to this lack of data for later time points, showed similar results. The moderators accounted for a relevant amount of heterogeneity (QM = 228.3, *df* = 20, *P* < 0.001), and there was a significant interaction TIME × ASSESSMENT [χ^2^(9) = 38.0, *P* < 0.001]. *Post-hoc*, significant changes over time were found for ARAT [χ^2^(1) = 16.5, β = 0.013, *P* < 0.001], BBT [χ^2^(1) = 5.2, β = 0.011, *P* = 0.023], F-M [χ^2^(1) = 6.3, β = 0.007, *P* = 0.012], GRIP [χ^2^(1) = 17.9, β = 0.023, *P* < 0.001], PEG [χ^2^(1) = 6.9, β = 0.017, *P* = 0.009], and RMA [χ^2^(1) = 4.1, β = 0.011, *P* = 0.043], but not for MI [χ^2^(1) = 0.9, *P* = 0.347], SIS [χ^2^(1) = 2.3, *P* = 0.127], or WMFT [χ^2^(1) = 2.3, *P* = 0.126] (Figure 2, Supplementary Table 6).

Overall, recovery was found to increase with the progression of time. To illustrate how recovery progressed in this current population across all assessments, the scores were merged into one figure (Supplementary Figure 3).

## Discussion

This work examines the predictive value of typically used assessments to measure arm motor recovery after stroke by comparing the extent to which a particular outcome measure quantified arm motor recovery. The metaregression highlighted four common assessments (ARAT, BBT, F-M, and GRIP) being capable of measuring motor recovery after stroke in a longitudinal fashion. After performing an LOCF analysis, also the PEG and RMA showed significant changes over 1 year. No such effects were found for WMFT, MI, and SIS. The latter can thus, based on the current data, not be recommended as longitudinal outcome measures of upper limb motor recovery in patients after a stroke.

To identify the most frequently used assessments while at the same time reducing the risk of selection bias, we identified studies with a large number of participants (*n* > 100), resulting in a sample, which is in line with other work on this topic (5). ARAT and F-M are common assessments in large-scale stroke trials and show similar progression slopes in our model predictions (Figure 2). They both require an experienced rater to ensure a rapid and reliable evaluation. In addition, there seems to be a ceiling effect, especially for F-M. The scale could most likely indicate changes in those patients with severe and moderate deficits who will not achieve the maximum possible score (60). GRIP, an objective and metric assessment of strength, has been included in numerous studies investigating recovery after stroke, but seldom in studies with follow-ups going beyond 3 months (61). Regarding clinicians working in stroke rehabilitation at hospital settings, the assessment GRIP, next to BBT, and PEG are already established (62). From the scales assessed, GRIP provided the most steady upward trend during rehabilitation as time progressed. In line with recommendations on measurement protocols concerning performance of body function after stroke (1), the current results can support the use of GRIP as assessment of hand function recovery, focusing on muscle strength. ARAT, F-M, BBT, and GRIP; all these scales were found to show a significant change over time in the initial data set. This result can be linked to, on the one hand, the availability of scores across the anticipated time frame and, on the other hand, the hypothesized potential of these outcome measures to quantify recovery over time reliably.

One initial concern was that releveling the original scale to a standardized ratio-scaled score could limit the potential of the individual assessments. This would primarily affect the RMA and MI. Other scales, where scores are based on time (BBT, WMFT) or other ratio scaled measures such as force (GRIP, PEG) or where a ratio scaled evaluation is intended (SIS), are unaffected by the transformation. The ARAT and F-M both do not provide ratio level scores, even though they are commonly treated as if they do. The MI did not show any significant change over time. As it intrinsically provides a score between 0 and 100, there was no need for any additional mathematical standardization. However, this score is solely based on the evaluation of three movements for the upper limb, but can be expanded by additional three movements for the lower limb. Its unique features enable a rapid evaluation on the one hand but may not be fine-grained enough for longitudinal evaluations. While the scale was initially also, in combination with other scales, designed to follow up on the evolution in time, the original scales evaluation did not go beyond 6 months poststroke (58). Malmut et al. (63) found the MI capable of predicting upper limb recovery, however, also not going beyond 3 months. Thus, while it may be a solid assessment for a snapshot evaluation of patients in daily clinical practice, it cannot be recommended to document long-term recovery paths. In contrast to the MI, the RMA incorporates 15 different scoring possibilities, which seems fine-grained enough to portray recovery over time.

The WMFT provides two different outcome measures. Here we chose to evaluate the performance time in seconds, as this was most commonly reported. It can be argued that performance time coheres more with motor compensation in contrast to movement quality, which may portray motor recovery better. Thus, movement quality may be the choice of outcome when it comes to long-term change. Alas, the lack of available data and gold standard leaves this question open to future research. Some have even chosen the F-M over the WMFT as primary outcome measure, as the F-M captures impairment and thus highlights the return to prestroke movement patterns compared to activity performance captured with the WMFT related to compensation (64).

The scales included in the present analysis are used in large stroke trials and comply with the consensus core recommendations (2). Other non-motor, pure-motor scores, not assessing motor recovery, such as specific scales classifying spasticity (e.g., Ashworth and Tardieu), were not included. Highly specialized scales for hand function (e.g., Jebsen–Taylor, etc.) could not be included as their use was not reported in the current sample of large stroke trials. It may well be that other scales not present in the current analysis are also very well capable of describing recovery over time.

### Limitations

The current research has limitations, mostly pertaining to intrinsic properties of meta-analyses and the heterogeneity of the underlying data. A meta-analysis as applied here summarizes averaged scores of different subpopulations and reduces unique features of individual participants to a more homogeneous group. This could be compensated for by an individual patient data–based meta-analysis [e.g., Thomalla et al. (65)]. However, we had no access to the individual data of all 4,433 patients. To account for this loss in heterogeneity, a random mixed-effects model was chosen over a mixed model, as it includes weights relative to the study population from which the information was drawn, making studies with a larger study population more influential on the relationship between moderators than smaller studies (66).

The second limitation concerns the sparsity of published rehabilitation progress following 6 months after stroke, and also, the time point “5 months after stroke” was rarely documented. Here we have to remark that we used only one database for literature search. Nevertheless, additional literature was identified by searching the reference list of the recent and comprehensive Cochrane Review by Pollock and colleagues (13). Thus, it is reasonable to expect that the largest studies, explicitly searched for our purpose, were included. The analysis was based on the assumption that the acquired data reflect a representative sample of stroke patients. However, with a reduction of data over time, this assumption may be suspended. We tried to account for this loss by including an LOCF analysis, therefore assuming that no further improvement will occur. This reflects clinical experience in later stages of the recovery process but does not necessarily align well with the assumption that recovery follows a natural logarithmic curve if values are missing at earlier time points after stroke. Both PEG and RMA only reached significance over time with the LOCF analysis, once more highlighting the detrimental effects of long-term data sparsity on the one hand and the need for additional longitudinal evaluation of especially these scales on the other.

Many studies included individuals with a broad time range after stroke. The mean time after stroke, relative to the current measure, had to be estimated based on the information provided by the respective authors. If recruitment took place from stroke onset until 6 months after stroke with a mean of 2 months after stroke [as in Adie et al. (32)], the baseline measure for the entire group had to be assumed to be 2 months after stroke. Additional data points were subsequently assigned to time points relative to baseline. This approach, performed in some, but not all studies, could result in a systematic error, study-wise. Some authors provided individual patient data and the respective time since stroke enabling us to retrospectively resort and regroup these patients according to the actual time poststroke. Other authors provided new summary tables where patients had already been resorted according to their respective time since stroke. And finally, some studies had very narrow inclusion criteria where no resorting was required. The current sample size of 4,433 patients is large. Thus, it can be assumed that it contains a representative, wide spectrum of patients, even though a selection bias across all studies cannot be ruled out completely, especially as very severely affected patients are rarely included. Next to the high burden to include severely affected patients in clinical trials, most of the aforementioned scales are not able to quantify arm function within this population. Additional research may be required to address outcome measures best for this specific population.

Even for studies focusing on upper limb recovery, finding a combination of all nine most common scales within one trial is highly unlikely. Common reasons would include the lack of time, the need to include further research-specific scales, and the general presumption that some of these scales may be redundant. The question of how the scales relate to one another can only be answered with a larger number of patient data, all measured with the same assessments at identical time points. For this reason, the current results do not allow deriving scores from one assessment to another, nor do they promote the discontinued use of other assessments.

### Conclusion

The current results may add to some issues discussed in the rehabilitation roundtable on consensus-based core recommendation (2). We found ARAT, BBT, F-M, GRIP, PEG, and RMA to be suitable instruments to document recovery after stroke; WMFT, MI, and SIS were less convincing in the longitudinal perspective. Interdependencies between different scales, which could make measuring multiple scales redundant, need to be considered and can best be analyzed with individual patient data. It should be a common goal to increase the comparability between stroke studies, where data acquisition is extraordinarily time-consuming and sometimes also stressful for the patient. Future research protocols should include multiple scales, preferably ARAT, BBT, F-M, GRIP, PEG, RMA, and, in line with the stroke recovery and rehabilitation roundtable consensus (2), a kinematic measure at identical or at least similar time points after stroke within one individual and over 1-year time. Also, especially large-scale trials should facilitate future meta-analyses and make data publicly available.

## Data Availability Statement

The raw data supporting the conclusions of this article will be made available by the authors, without undue reservation.

## Author Contributions

SW performed the article screening and methodological assessment, discussed the results, and prepared the manuscript. CG discussed the results and prepared the manuscript. WB provided the initial research idea, performed the article screening and the statistical analysis, discussed the results, and prepared the manuscript. All authors contributed significantly.

## Conflict of Interest

The authors declare that the research was conducted in the absence of any commercial or financial relationships that could be construed as a potential conflict of interest.
