# Supplementary material for: Predictive Value of Upper Extremity Outcome Measures After Stroke—A Systematic Review and Metaregression Analysis
Source: Front Neurol. 2021 Jun 10;12:675255. doi: 10.3389/fneur.2021.675255 (PMC8222610; doi:10.3389/fneur.2021.675255)
Supplement: Supplementary file 1 [file Data_Sheet_1.docx]

Supplementary Material

# Supplementary Data

## Initial search Query

(((((((((((("stroke") OR cerebral stroke[MeSH Terms]) OR "cerebrovascular stroke") OR cerebrovascular stroke[MeSH Terms]) OR cerebrovascular accident[MeSH Terms]) OR "apoplexy") OR apoplexy[MeSH Terms]) OR apoplexy, cerebrovascular[MeSH Terms]))))

AND

((((("upper limb") OR upper limb[MeSH Terms]) OR "upper extremity") OR upper extremity[MeSH Terms]))) AND ((Clinical Study[ptyp] OR Clinical Trial[ptyp] OR Observational Study[ptyp] OR Randomized Controlled Trial[ptyp]) AND ("2013/01/01"[PDat] : "2018/05/04"[PDat])

AND

(English[lang] OR German[lang]))

## Follow-up search Query

(((((((((((("stroke") OR cerebral stroke[MeSH Terms]) OR "cerebrovascular stroke") OR cerebrovascular stroke[MeSH Terms]) OR cerebrovascular accident[MeSH Terms]) OR "apoplexy") OR apoplexy[MeSH Terms]) OR apoplexy, cerebrovascular[MeSH Terms]))))

AND

((((("upper limb") OR upper limb[MeSH Terms]) OR "upper extremity") OR upper extremity[MeSH Terms]))) AND ((Clinical Study[ptyp] OR Clinical Trial[ptyp] OR Observational Study[ptyp] OR Randomized Controlled Trial[ptyp]) AND ("2013/01/01"[PDat] : "2020/11/13"[PDat])

AND

(English[lang] OR German[lang]))

# Supplementary Figures and Tables

## Supplementary Figures


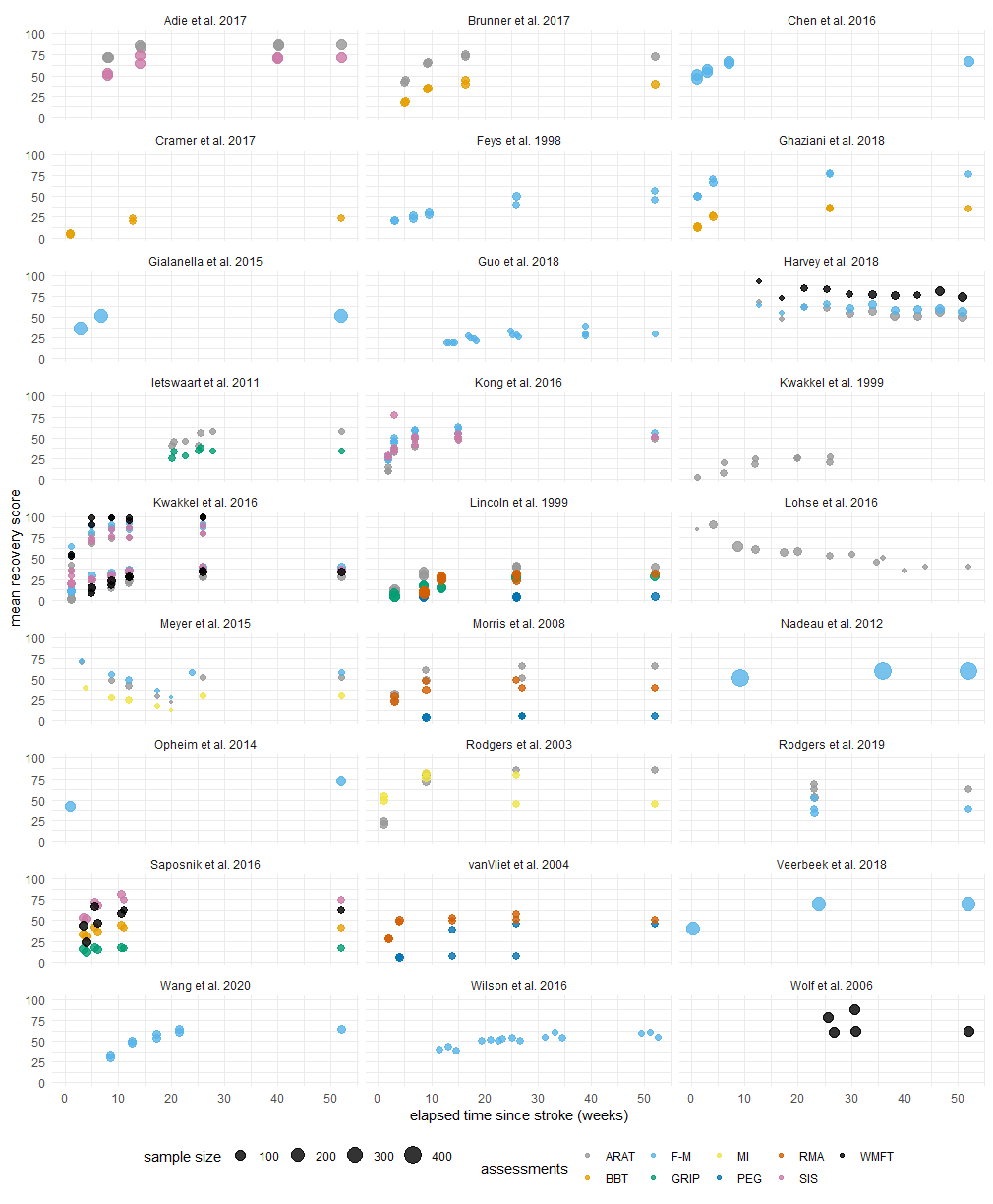


**Supplementary Figure 1.** Transformed data from individual data sources in percentage of recovery over the time course of one year after stroke. Individual assessments are color coded and the size depicts the underlying sample size. ARAT – Action research Arm Test, BBT – Box and Block test, F-M – Fugl Meyer Assessment (upper extremity), GRIP – Grip force, MI – Motricity Index, PEG – Peg test, RMA – Rivermead Motor Assessment, SIS – Stroke Impact Scale, WMFT – Wolf Motor Function Test.


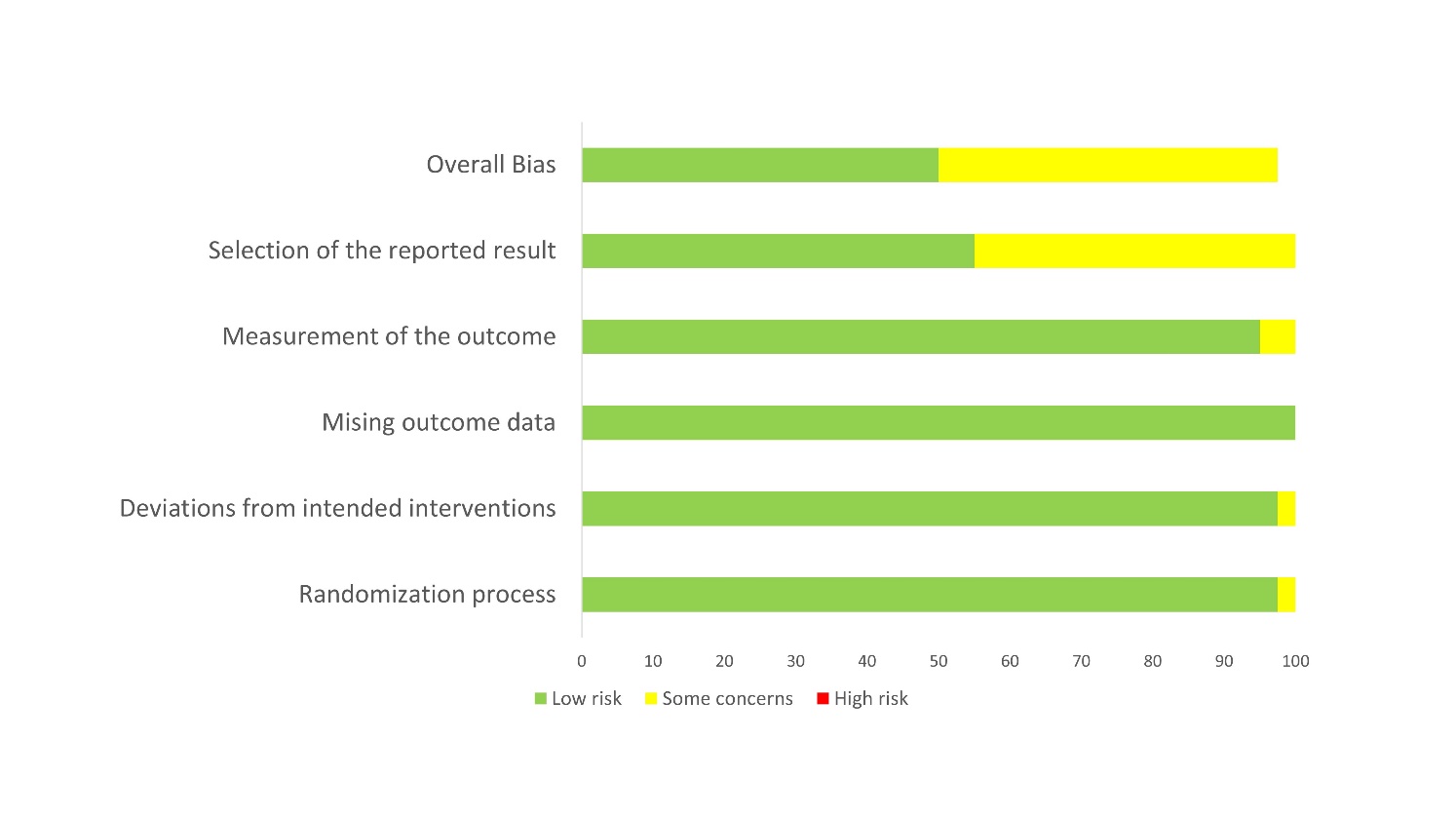


**Supplementary Figure 2.** Risk of bias graph - methodological quality of included randomized trials rated with the Cochrane Risk of Bias Tool RoB2.


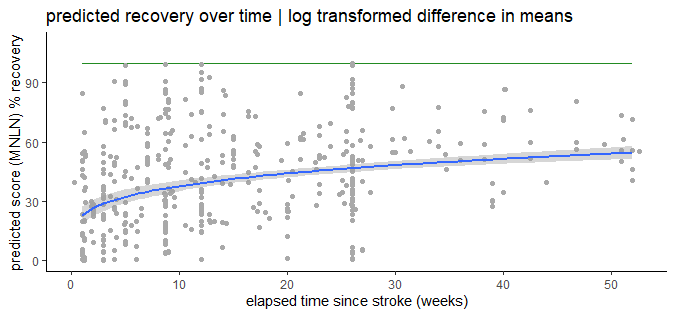
**Supplementary Figure 3.** Prediction of recovery across all assessments over the time course of one year cross-fading raw values. The light-green line resembles a healthy score, respective to 100% recovery

## Supplementary Tables

| contacted authors for additional information | extracted data from plots | recalculated means and sd from median and range | dropped reference due to missing information |
| --- | --- | --- | --- |
| Adie et al. 2017  Feys et al. 1998  Gialanella et al. 2015  Ghaziani et al. 2018  Harvey et al. 2018  Kong et al. 2016  Kwakkel et al. 1999  Lang et al. 2013  Lincoln et al. 1999  Lohse et al. 2016  Meyer et al. 2015  Nadeau et al. 2014  Persson et al. 2015  Rodgers et al. 2003  Rodgers et al. 2019  Saposnik et al. 2016  Shaw et al. 2010  Stinear et al. 2017  Winstein et al. 2016  Wolf et al. 2006  Zheng et al. 2015 | Feys et al. 1998 FMA  Wolf et al. 2006 WMFT | Kwakkel et al. 1999 ARAT | Feys et al. 1998 ARAT  Kwakkel et al. 1999 FMA  Saposnik et al. 2016 WMFT, Grip, SIS |

**Supplementary Table 1.** Overview over studies of which the original articles did not provide sufficient information for the meta-regression or where data was extracted by other means and studies dropped from the final analysis.

|  | **N ischemic infarction** | **N Dominant hand = right** | **N Affected arm = right** | **N Affected arm = dominant side** |
| --- | --- | --- | --- | --- |
| Adie et al. 2017 (1) | 209 | 206 | * | * |
| Brunner et al. 2017 (2) | 95 | 108 | 53 | * |
| Chen et al. 2016 (3) | 250 | * | 88 | * |
| Cramer et al. 2017(4) | 133 | * | * | * |
| Feys et al. 1998 (5) | 95 | * | 42 | * |
| Ghaziani et al. 2018 (6) | 80 | 100 | 53 | * |
| Gialanella & Santoro 2015 (7) | 167 | * | * | * |
| Guo et al. 2019 (8) | 54 | * | * | * |
| Harvey et al. 2018 (9) | 157 | * | 94 | 178 |
| Ietswaart et al. 2011 (10) | * | * | 52 | * |
| Kong et al. 2016 (11) | 80 | * | 40 | * |
| Kwakkel et al. 1999 (12) | * | * | * | * |
| Kwakkel et al. 2016 (13) | 159 | * | 54 | 54 |
| Lincoln et al. 1999 (14) | * | 282 | 125 |  |
| Lohse et al. 2016 (15) | * | * | * | * |
| Meyer et al. 2016 (16) | 108 | 113 | 48 | * |
| Morris et al. 2008 (17) | 9 | 54 | 52 | 92 |
| Nadeau et al. 2014 (18) | 163 | * | * | * |
| Opheim et al. 2014 (19) | 100 | * | 55 | * |
| Rodgers et al. 2003 (20) | 112 | 111 | 54 | * |
| Rodgers et al. 2019 (21) | * | * | * | * |
| Saposnik et al. 2016 (22) | 141 | 125 | 66 | * |
| van Vliet et al. 2005 (23) | * | * | 61 | * |
| Veerbeek et al. 2018 (24) | 202 | * | 85 | * |
| Wang et al. 2020 (25) | 0 | * | * | * |
| Wilson et al. 2016 (26) | 98 | * | 62 | 41 |
| Wolf et al. 2006 (27) | 195 | * | * | 110 |

**Supplementary Table 2.** Additional background information on type of infarction, hemispherical dominance and handedness of the study population. *no information on this criteria was available.

| **Study ID** |  | **0** | **1** | **2** | **3** | **4** | **5** | **6** | **7** | **8** | **9** | **10** | **11** | **12** |
| --- | --- | --- | --- | --- | --- | --- | --- | --- | --- | --- | --- | --- | --- | --- |
| Adie et al. 2017 (1) |  |  |  | 0 |  | 11 |  |  |  |  |  | 15 |  |  |
| Brunner et al. 201 7(2) |  |  | 0 | 7 |  | 15 |  |  |  |  |  |  |  |  |
| Chen et al. 2016 (3) |  | 0 | 4 | 4 |  |  |  |  |  |  |  |  |  |  |
| Cramer et al. 2017 (4) |  | 0 |  |  | 39 |  |  |  |  |  |  |  |  |  |
| Feys et al. 1998 (5) |  |  | 0 | 0 | 0 |  |  | 4 |  |  |  |  |  | 10 |
| Ghaziani et al. 2018 (6) |  | 0 | 4 |  |  |  |  | 14 |  |  |  |  |  |  |
| Gialanella & Santoro 2015 (7) |  |  | 0 | 0 |  |  |  |  |  |  |  |  |  |  |
| Guo et al. 2018 (8) |  |  |  |  | 0 | 0 |  | 0 |  |  | 0 |  |  |  |
| Harvey et al. 2018 (9) * |  |  |  |  | 86 | 82 | 51 | 50 | 40 | 29 | 25 | 40 | 0 | 11 |
| Ietswaart et al. 2011 (10) |  |  |  |  |  |  | 0 | 0 |  |  |  |  |  |  |
| Kong et al. 2016 (11) |  | 0 | 0 | 3 |  | 5 |  |  |  |  |  |  |  |  |
| Kwakkel et al. 1999 (12) |  | 0 |  | 0 | 0 |  | 0 | 0 |  |  |  |  |  |  |
| Kwakkel et al. 2016 (13) |  | 0 | 3 | 3 | 3 |  |  | 3 |  |  |  |  |  |  |
| Lincoln et al. 1999 (14) |  |  | 20 | 0 | 51 |  |  | 23 |  |  |  |  |  |  |
| Lohse et al. 2016 (15) * |  | 96 | 43 | 0 | 35 | 35 | 49 | 62 | 78 | 79 | 86 | 87 | 86 | 85 |
| Meyer et al. 2016 (16) * |  |  | 74 | 31 | 0 | 62 | 87 | 33 |  |  |  |  |  |  |
| Morris et al. 2008 (16) |  |  | 0 | 8 |  |  |  |  | 20 |  |  |  |  |  |
| Nadeau et al. 2014 (18) |  |  |  | 0 |  |  |  |  |  |  | 0 |  |  |  |
| Opheim et al. 2014 (19) |  | 0 |  |  |  |  |  |  |  |  |  |  |  | 33 |
| Rodgers et al. 2003 (20) |  | 0 |  | 15 |  |  |  | 41 |  |  |  |  |  |  |
| Rodgers et al. 2019 (21) |  |  |  |  |  |  | 0 |  |  |  |  |  |  |  |
| Saposnik et al. 2016 (22) |  |  | 0 | 14 | 30 |  |  |  |  |  |  |  |  |  |
| van Vliet et al. 2005 (23) |  | 39 | 0 |  | 14 |  |  | 12 |  |  |  |  |  |  |
| Veerbeek et al. 2018 (24) |  | 0 |  |  |  |  |  | 0 |  |  |  |  |  |  |
| Wang et al. 2020 (25) |  |  |  | 0 | 0 | 0 | 0 |  |  |  |  |  |  |  |
| Wilson et al. 2016 (26) |  |  |  |  | 0 |  | 13 | 17 |  | 25 |  |  |  | 30 |
| Wolf et al. 2006 (27) |  |  |  |  |  |  |  | 0 | 8 |  |  |  |  |  |

**Supplementary Table 3.** Drop-out rate per time point and included study in percentage. When multiple assessments were measured per study, the drop-out rate was averaged over all measured assessments. Studies marked by an asterix (*) provided individual patient data, thus the drop-out rate is relative to the largest sample size provided at any time-point and not representative for the provided data set.

| **Month** | **0** | **1** | **2** | **3** | **4** | **5** | **6** | **7** | **8** | **9** | **10** | **11** | **12** |
| --- | --- | --- | --- | --- | --- | --- | --- | --- | --- | --- | --- | --- | --- |
| **Total** | 2416 | 4019 | 5019 | 2571 | 1321 | 1007 | 3294 | 634 | 296 | 736 | 566 | 272 | 496 |
| **Participants** | 1510 | 1814 | 3156 | 1652 | 887 | 759 | 2077 | 464 | 231 | 736 | 465 | 272 | 496 |
| **Study groups** | 46 | 70 | 78 | 57 | 30 | 27 | 68 | 12 | 7 | 9 | 8 | 4 | 10 |
| **Studies** | 11 | 13 | 16 | 13 | 8 | 8 | 15 | 4 | 3 | 4 | 3 | 2 | 5 |

**Supplementary Table 4.** Overview of available data per time point. ‘Total’ represent the summed number of collected measures from participants per study and per assessment. ‘Participants’ depicts the total number of participants per time point. ‘Study groups’ summarize the number of different study groups (f.i. treatment, control) per time point over all studies, and ‘Studies’ represents the number of studies of whom data points have been collected during the respective month after stroke.

| **Month** | **0** | **1** | **2** | **3** | **4** | **5** | **6** | **7** | **8** | **9** | **10** | **11** | **12** |
| --- | --- | --- | --- | --- | --- | --- | --- | --- | --- | --- | --- | --- | --- |
| **ARAT** | 13 | 16 | 23 | 10 | 10 | 12 | 17 | 4 | 2 | 2 | 4 | 2 | 2 |
| **BBT** | 4 | 6 | 4 | 4 | 2 | 0 | 2 | 0 | 0 | 0 | 0 | 0 | 0 |
| **FMA** | 13 | 15 | 16 | 17 | 11 | 10 | 18 | 1 | 4 | 6 | 1 | 1 | 7 |
| **grip force** | 1 | 6 | 5 | 5 | 0 | 3 | 7 | 0 | 0 | 0 | 0 | 0 | 0 |
| **Motricity** | 2 | 1 | 3 | 1 | 1 | 1 | 3 | 0 | 0 | 0 | 0 | 0 | 0 |
| **peg test** | 0 | 7 | 5 | 2 | 0 | 0 | 5 | 2 | 0 | 0 | 0 | 0 | 0 |
| **Rivermead** | 2 | 4 | 5 | 5 | 0 | 0 | 5 | 2 | 0 | 0 | 0 | 0 | 0 |
| **SIS** | 7 | 9 | 11 | 6 | 5 | 0 | 4 | 0 | 0 | 0 | 2 | 0 | 0 |
| **WMFT** | 4 | 6 | 6 | 7 | 1 | 1 | 7 | 3 | 1 | 1 | 1 | 1 | 1 |
| **sum** | 46 | 70 | 78 | 57 | 30 | 27 | 68 | 12 | 7 | 9 | 8 | 4 | 10 |

**Supplementary Table 5.** Data points per time point. Each study group is counted as an individual data point per assessment and time point. Month 0 corresponds to assessment measured within the first two weeks after stroke.

| Assessment | Time After Stroke | Recovery (%) | Original Scale |
| --- | --- | --- | --- |
| ARAT | 3 months (12 weeks) | 43.6 (27.8,68.3) | 24.9 (15.9,38.9) |
|  | 6 months (24 weeks) | 50.9 (32.5,79.6) | 29 (18.5,45.4) |
|  | 12 months (52 weeks) | 57.8 (36.1,92.6) | 32.9 (20.5,52.8) |
| BBT | 3 months (12 weeks) | 27.6 (17.1,44.3) | 20.4 (12.7,32.8) |
|  | 6 months (24 weeks) | 31.5 (19.5,50.8) | 23.3 (14.4,37.6) |
|  | 12 months (52 weeks) | 34 (19.3,60.1) | 25.2 (14.2,44.5) |
| F-M | 3 months (12 weeks) | 52.2 (33.4,81.7) | 34.5 (22,53.9) |
|  | 6 months (24 weeks) | 56.8 (36.3,88.8) | 37.5 (24,58.6) |
|  | 12 months (52 weeks) | 54.6 (34.3,86.9) | 36.1 (22.7,57.4) |
| GRIP | 3 months (12 weeks) | 19.4 (12,31.1) | 19.4 (12,31.1) |
|  | 6 months (24 weeks) | 25.5 (15.9,41.1) | 25.5 (15.9,41.1) |
|  | 12 months (52 weeks) | 38.6 (21.5,69.2) | 38.6 (21.5,69.2) |
| healthy | 3 months (12 weeks) | 112.8 (72.1,176.5) | 112.8 (72.1,176.5) |
|  | 6 months (24 weeks) | 113.1 (72.4,176.5) | 113.1 (72.4,176.5) |
|  | 12 months (52 weeks) | 90.1 (57.1,142.1) | 90.1 (57.1,142.1) |
| MI | 3 months (12 weeks) | 45 (27,75.1) | 45 (27,75.1) |
|  | 6 months (24 weeks) | 41.8 (25.1,69.6) | 41.8 (25.1,69.6) |
|  | 12 months (52 weeks) | 27.9 (14.5,53.7) | 27.9 (14.5,53.7) |
| PEG | 3 months (12 weeks) | 6.1 (3.7,10.1) | 0.1 (0.1,0.2) |
|  | 6 months (24 weeks) | 7.5 (4.6,12.2) | 0.2 (0.1,0.3) |
|  | 12 months (52 weeks) | 9.6 (5.3,17.5) | 0.2 (0.1,0.4) |
| RMA | 3 months (12 weeks) | 33.3 (20.6,54) | 5 (3.1,8.1) |
|  | 6 months (24 weeks) | 38 (23.5,61.4) | 5.7 (3.5,9.2) |
|  | 12 months (52 weeks) | 40.9 (23,72.7) | 6.1 (3.4,10.9) |
| SIS | 3 months (12 weeks) | 53.6 (33.9,84.8) | 13.4 (8.5,21.2) |
|  | 6 months (24 weeks) | 58 (36.5,92.2) | 14.5 (9.1,23.1) |
|  | 12 months (52 weeks) | 55.2 (32.2,94.4) | 13.8 (8.1,23.6) |
| WMFT | 3 months (12 weeks) | 61.9 (38.7,98.8) | 46.6 (74,2.7) |
|  | 6 months (24 weeks) | 67.5 (42.4,107.2) | 39.9 (69.6,-7.2) |
|  | 12 months (52 weeks) | 65.4 (38.5,111.2) | 42.3 (74.3,-12) |

**Supplementary Table 6.** Predicted recovery scores. The final model (LOCF) indicated a significant change in measured recovery after stroke over time when measured with the ARAT, BBT, F-M, GRIP, PEG, or when measuring RMA. The table presents predicted values within the standardized scale and in the original scale.

REFERENCES

1. Adie K, Schofield C, Berrow M, Wingham J, Humfryes J, Pritchard C, et al. Does the use of Nintendo Wii SportsTM improve arm function? Trial of WiiTM in Stroke: a randomized controlled trial and economics analysis. *Clin Rehabil* (2017) **31**:173–85. doi:10.1177/0269215516637893

2. Brunner I, Skouen JS, Hofstad H, Aßmus J, Becker F, Sanders A-M, et al. Virtual Reality Training for Upper Extremity in Subacute Stroke (VIRTUES): A multicenter RCT. *Neurology* (2017) **89**:2413–21. doi:10.1212/WNL.0000000000004744

3. Chen L, Fang J, Ma R, Gu X, Chen L, Li J, et al. Additional effects of acupuncture on early comprehensive rehabilitation in patients with mild to moderate acute ischemic stroke: a multicenter randomized controlled trial. *BMC Complement Altern Med* (2016) **16**:226. doi:10.1186/s12906-016-1193-y

4. Cramer SC, Enney LA, Russell CK, Simeoni M, Thompson TR. Proof-of-Concept Randomized Trial of the Monoclonal Antibody GSK249320 Versus Placebo in Stroke Patients. *Stroke* (2017) **48**:692–8. doi:10.1161/STROKEAHA.116.014517

5. Feys HM, Weerdt WJ de, Selz BE, Cox Steck GA, Spichiger R, Vereeck LE, et al. Effect of a therapeutic intervention for the hemiplegic upper limb in the acute phase after stroke: a single-blind, randomized, controlled multicenter trial. *Stroke* (1998) **29**:785–92. doi:10.1161/01.str.29.4.785

6. Ghaziani E, Couppé C, Siersma V, Søndergaard M, Christensen H, Magnusson SP. Electrical Somatosensory Stimulation in Early Rehabilitation of Arm Paresis After Stroke: A Randomized Controlled Trial. *Neurorehabil Neural Repair* (2018) **32**:899–912. doi:10.1177/1545968318799496

7. Gialanella B, Santoro R. Prediction of functional outcomes in stroke patients: the role of motor patterns according to limb synergies. *Aging Clin Exp Res* (2015) **27**:637–45. doi:10.1007/s40520-015-0322-7

8. Guo J, Qian S, Wang Y, Xu A. Clinical study of combined mirror and extracorporeal shock wave therapy on upper limb spasticity in poststroke patients. *Int J Rehabil Res* (2019) **42**:31–5. doi:10.1097/MRR.0000000000000316

9. Harvey RL, Edwards D, Dunning K, Fregni F, Stein J, Laine J, et al. Randomized Sham-Controlled Trial of Navigated Repetitive Transcranial Magnetic Stimulation for Motor Recovery in Stroke. *Stroke* (2018) **49**:2138–46. doi:10.1161/STROKEAHA.117.020607

10. Ietswaart M, Johnston M, Dijkerman HC, Joice S, Scott CL, MacWalter RS, et al. Mental practice with motor imagery in stroke recovery: randomized controlled trial of efficacy. *Brain* (2011) **134**:1373–86. doi:10.1093/brain/awr077

11. Kong K-H, Loh Y-J, Thia E, Chai A, Ng C-Y, Soh Y-M, et al. Efficacy of a Virtual Reality Commercial Gaming Device in Upper Limb Recovery after Stroke: A Randomized, Controlled Study. *Top Stroke Rehabil* (2016) **23**:333–40. doi:10.1080/10749357.2016.1139796

12. Kwakkel G, Wagenaar RC, Twisk JW, Lankhorst GJ, Koetsier JC. Intensity of leg and arm training after primary middle-cerebral-artery stroke: a randomised trial. *Lancet* (1999) **354**:191–6. doi:10.1016/S0140-6736(98)09477-X

13. Kwakkel G, Winters C, van Wegen EE, Nijland RH, van Kuijk AA, Visser-Meily A, et al. Effects of Unilateral Upper Limb Training in Two Distinct Prognostic Groups Early After Stroke: The EXPLICIT-Stroke Randomized Clinical Trial. *Neurorehabil Neural Repair* (2016) **30**:804–16. doi:10.1177/1545968315624784

14. Lincoln NB, Parry RH, Vass CD. Randomized, controlled trial to evaluate increased intensity of physiotherapy treatment of arm function after stroke. *Stroke* (1999) **30**:573–9. doi:10.1161/01.str.30.3.573

15. Lohse K, Bland MD, Lang CE. Quantifying Change During Outpatient Stroke Rehabilitation: A Retrospective Regression Analysis. *Arch Phys Med Rehabil* (2016) **97**:1423-1430.e1. doi:10.1016/j.apmr.2016.03.021

16. Meyer S, Bruyn N de, Lafosse C, van Dijk M, Michielsen M, Thijs L, et al. Somatosensory Impairments in the Upper Limb Poststroke: Distribution and Association With Motor Function and Visuospatial Neglect. *Neurorehabil Neural Repair* (2016) **30**:731–42. doi:10.1177/1545968315624779

17. Morris JH, van Wijck F, Joice S, Ogston SA, Cole I, MacWalter RS. A comparison of bilateral and unilateral upper-limb task training in early poststroke rehabilitation: a randomized controlled trial. *Arch Phys Med Rehabil* (2008) **89**:1237–45. doi:10.1016/j.apmr.2007.11.039

18. Nadeau SE, Lu X, Dobkin B, Wu SS, Dai YE, Duncan PW. A prospective test of the late effects of potentially antineuroplastic drugs in a stroke rehabilitation study. *Int J Stroke* (2014) **9**:449–56. doi:10.1111/j.1747-4949.2012.00920.x

19. Opheim A, Danielsson A, Alt Murphy M, Persson HC, Sunnerhagen KS. Upper-limb spasticity during the first year after stroke: stroke arm longitudinal study at the University of Gothenburg. *Am J Phys Med Rehabil* (2014) **93**:884–96. doi:10.1097/PHM.0000000000000157

20. Rodgers H, Mackintosh J, Price C, Wood R, McNamee P, Fearon T, et al. Does an early increased-intensity interdisciplinary upper limb therapy programme following acute stroke improve outcome? *Clin Rehabil* (2003) **17**:579–89. doi:10.1191/0269215503cr652oa

21. Rodgers H, Bosomworth H, Krebs HI, van Wijck F, Howel D, Wilson N, et al. Robot assisted training for the upper limb after stroke (RATULS): a multicentre randomised controlled trial. *Lancet* (2019) **394**:51–62. doi:10.1016/S0140-6736(19)31055-4

22. Saposnik G, Cohen LG, Mamdani M, Pooyania S, Ploughman M, Cheung D, et al. Efficacy and safety of non-immersive virtual reality exercising in stroke rehabilitation (EVREST): a randomised, multicentre, single-blind, controlled trial. *The Lancet Neurology* (2016) **15**:1019–27. doi:10.1016/S1474-4422(16)30121-1

23. van Vliet PM, Lincoln NB, Foxall A. Comparison of Bobath based and movement science based treatment for stroke: a randomised controlled trial. *J Neurol Neurosurg Psychiatry* (2005) **76**:503–8. doi:10.1136/jnnp.2004.040436

24. Veerbeek JM, Winters C, van Wegen EE, Kwakkel G. Is the proportional recovery rule applicable to the lower limb after a first-ever ischemic stroke? *PLoS ONE* (2018) **13**:e0189279. doi:10.1371/journal.pone.0189279

25. Wang H-Q, Hou M, Li H, Bao C-L, Min L, Dong G-R, et al. Effects of acupuncture treatment on motor function in patients with subacute hemorrhagic stroke: A randomized controlled study. *Complement Ther Med* (2020) **49**:102296. doi:10.1016/j.ctim.2019.102296

26. Wilson RD, Page SJ, Delahanty M, Knutson JS, Gunzler DD, Sheffler LR, et al. Upper-Limb Recovery After Stroke: A Randomized Controlled Trial Comparing EMG-Triggered, Cyclic, and Sensory Electrical Stimulation. *Neurorehabil Neural Repair* (2016) **30**:978–87. doi:10.1177/1545968316650278

27. Wolf SL, Winstein CJ, Miller JP, Taub E, Uswatte G, Morris D, et al. Effect of constraint-induced movement therapy on upper extremity function 3 to 9 months after stroke: the EXCITE randomized clinical trial. *JAMA* (2006) **296**:2095–104. doi:10.1001/jama.296.17.2095
